# Concomitant lorazepam use and antidepressive efficacy of repetitive transcranial magnetic stimulation in a naturalistic setting

**DOI:** 10.1007/s00406-020-01160-9

**Published:** 2020-07-09

**Authors:** M. Deppe, M. Abdelnaim, T. Hebel, P. M. Kreuzer, T. B. Poeppl, B. Langguth, M. Schecklmann

**Affiliations:** 1 Department of Psychiatry and Psychotherapy, University Regensburg, Regensburg, Germany; 2 Department of Psychiatry, Psychotherapy, and Psychosomatics, Medical Faculty, RWTH Aachen University, Aachen, Germany

**Keywords:** rTMS, Brain stimulation, Depression, Affective disorder, Lorazepam, Benzodiazepine

## Abstract

**Background/objectives:**

Repetitive transcranial magnetic stimulation (rTMS) has been established as an effective therapeutic intervention for the treatment of depression. Preliminary data suggest that the efficacy of rTMS is reduced in patients taking benzodiazepines (BZD). Here, we use real-world data from a large sample to investigate the influence of lorazepam on the effectiveness of rTMS.

**Methods:**

From a retrospective cohort of clinically depressed patients that were treated with rTMS, we compared 176 patients not taking any BZD with 73 patients taking lorazepam with respect to changes in the Hamilton Depression Rating Scale (HRDS).

**Results:**

Both groups improved during rTMS according to HRDS scores, but the amelioration of symptoms was significantly less pronounced in patients taking lorazepam (18% vs. 38% responders in the non-lorazepam group). We could not see any association of intake regimen of lorazepam with response in rTMS.

**Conclusion:**

Our observational study suggests that intake of lorazepam impedes the response to rTMS. The impact of lorazepam and other BZD on rTMS should receive more attention and be further investigated in prospective, hypothesis-based treatment studies to determine causal relationships between medication treatments and outcome. This could lead to specific recommendations for pharmacological treatment for depressed patients undergoing rTMS.

## Introduction

Depression is a psychiatric illness of high prevalence and it is associated with a high level of individual suffering [1, 2] as well as socioeconomic burden [3–5]. Depressive disorders are a heterogeneous group [1] of different disease entities, whose neurobiological pathophysiology is not yet well understood and controversially discussed [6–8]. Despite new pharmacological [8–10] and non-pharmacological [11] approaches, treatment outcome remains unsatisfactorily in a substantial proportion of patients [12, 13]. Repetitive transcranial magnetic stimulation (rTMS) is becoming increasingly important as an effective treatment method for depression with a few side effects and good patient acceptance in everyday clinical practice [14–24].

To further increase effectiveness of rTMS, a better understanding of interaction between rTMS and drugs is important*,* especially as most depressed individuals that are treated with rTMS also receive psychotropic medication. Despite their unfavorable side effect profile, especially in long-term use [25, 26], benzodiazepines (BZD) are widely used in the treatment of depression due to their good efficacy and rapid onset of action [25] with lorazepam being one of the most commonly prescribed BZD [27, 28]. Therefore, the influence of lorazepam on the antidepressive efficacy of rTMS is of particular interest.

A few clinical studies have investigated the interaction of BZD and rTMS in the treatment of depression. Hunter et al. [29] analyzed in a retrospective study 181 patients with MDD who were treated with rTMS and medication for 6 weeks to examine potential relationships between categories of medication use and clinical outcome to rTMS treatment for depression. Patients treated with a BZD showed a lower improvement at week 2 and a lower responder rate at week 6 compared to patients not treated with a BZD. In contrast, patients receiving psychostimulants showed a greater improvement at week 2 and over the entire 6 weeks, and a higher response rate than patients not receiving psychostimulants. All other drug groups studied did not affect the effect of rTMS treatment [29].

Kaster and colleagues reported a secondary analysis of the THREE-D study, a big randomized, multicentre, non-inferiority clinical trial [30], and could demonstrate that treatment response to rTMS is reduced in patients taking BZD [31]. This analysis raised awareness of the importance of the investigation of rTMS–medication interactions [32].

Caulfield and Stern retrospectively examined 58 patients with depression who were treated with rTMS and medication. An attempt was made to differentiate between rTMS effects on depression and on anxiety. rTMS was effective in treating both depression and anxiety regardless of the medication given. BZD users were pre- and post-treatment significantly more anxious than non-BZD users. Only in patients using BZD, changes in mood and anxiety were correlated. The authors themselves discussed a floor effect for non-BZD users as they started from lower anxiety scores [33].

In sum, studies investigating influence of BZD on rTMS effects in depression are in controlled trials but not with the aim of the study to investigate the effects of BZD. Data were analyzed retrospectively. In this study, we analyzed a large real-world sample of clinically depressed individuals receiving rTMS treatment under naturalistic conditions (high ecological validity) with the question whether concomitant use of lorazepam, one of the most commonly used BZD [27, 28], influences the antidepressive effectiveness of rTMS.

## Methods

From a retrospective cohort of 716 patients with depression who were treated with rTMS in the Center for Neuromodulation Regensburg (Germany) between 2002 and 2017, complete data of the Hamilton Depression Rating Scale (HDRS; 44) at begin and end of treatment were available for 299 patients [23].

Response was defined as reduction of the HDRS-17 score by 50% or more at the end of treatment as compared to baseline.

The inclusion criteria for this retrospective analysis were naïvity to rTMS (only the patient’s first treatment with rTMS was considered), clinical depression with diagnosis F31–F33 according to ICD-10 (single or recurrent episode of unipolar or bipolar depression, each with or without psychotic symptoms), a complete documented HDRS at beginning and at the end the rTMS treatment and absence of any serious somatic illness. Both in- and outpatients were included. Patients were grouped according to their intake of BZD and z-drugs (zopiclone or zolpidem). Seventy-three patients took only lorazepam (group lorazepam). Fifty patients took other BZD or z-drugs (30 with z-drugs, six with oxazepam, one with lorametazepam, two with bromazepam, and two with z-drugs and BZD) and 176 took no BZD or z-drug (group no BZD). For the analysis, we concentrated on the groups lorazepam and no BZD as the scope of the paper was BZD and not z-drugs (due to differences in use) and the number of other BZDs were too small for adequate analyses.

Patients were treated with different rTMS protocols including stimulation of the left, right, bilateral dorsolateral, and also dorsomedial prefrontal cortex [23, 34–36]. There were no significant differences in the antidepressant effect of rTMS between different treatment protocols (*χ*^2^ = 8.569; df = 7; *p* = 0.285). A typical treatment lasted two up to 6 weeks, whereas in individual cases, treatment duration was different. No accelerated protocols were applied.

All data were analyzed using SPSS (IBM Corp., USA; Version 24.0.0.0). The significance level was set at *p* < 0.05 for group contrasts. For group contrasts we used Student’s *t* tests or Chi-square test of independence depending on the scales of measurement. Effect sizes for HDRS group contrasts were reported by Cohen’s d [37]. To exclude an influence of the baseline HDRS values on the group differences evaluated with *T* tests, we analyzed changes from pre- to post-treatment for the two groups by an analysis of covariance with the within-subjects factor rTMS (pre- and post-rTMS) and the between-subjects factor group (no BZD or z-drug vs. lorazepam) and the covariate baseline HDRS score for the dependent variables HDRS-21 and HDRS-17. This affirmed the results of the group comparisons.

To evaluate the influence of dosage regimen of lorazepam on rTMS effects, we correlated the sum of taken lorazepam over all days, the number of days lorazepam was taken, the average daily dosage, the relative proportion of the number of days lorazepam was taken to the number of rTMS treatment days, and the relation of the sum of lorazepam to the number of rTMS treatment days using Pearson correlation coefficients. We also compared the groups with an average daily dosage of maximal 1 mg and above 1 mg by Student’s *t* tests.

## Results

Both groups did not differ with respect to age, sex, resting motor threshold, stimulation intensity, number of treatment sessions, number of pulses per session, type and severity of depression, and baseline depressive symptoms (HDRS) (Table 1). Overall patients showed an amelioration of symptoms with rTMS, which was significantly less pronounced in patients taking lorazepam as compared to those who did not take BZD (Table1). 18% of the patients with lorazepam were responders (> 50% decrease in symptoms in HDRS-17) in contrast to 38% of the patients without lorazepam. Effect sizes were small for group contrasts. We could not show an association of the absolute sum of lorazepam dosage over the course of treatment (all 0.114 ≤ *r* ≤ 0.193; all *p* values ≥ 0.102) and relative to the number of treatment days (all 0.055 ≤ *r* ≤ 0.140; all *p* values ≥ 0.237), the absolute (all 0.146 ≤ *r* ≤ 0.202; all *p* values ≥ 0.087) or relative number of days taking lorazepam (all 0.051 ≤ r ≤ 0.115; all *p* values ≥ 0.332) or the daily dosage of lorazepam with changes in HDRS scores (all 0.070 ≤ *r* ≤ 0.148; all *p* values ≥ 0.212). Dividing the group into patients with daily dosage over and below 1 mg did not show significant differences (all *T* values ≤ 1.534; df = 71; all *p* values ≥ 0.129). In sum, the fact that lorazepam is taken lowers the response to rTMS without association of intake regimen of lorazepam in relation to rTMS.

**Table 1:** Characteristics of patients with depression taking vs. not-taking lorazepam

	No BZD or Z-drug (*n* = 176)	Lorazepam (*n* = 73)	Statistics for group contrasts
Age (years)	47.8 ± 12.3	49.3 ± 13.3	*T* = 0.809, df = 247, p = 0.420
Sex (female/male)	91/85	46/27	*χ*^2^ = 2.667, df = 1, *p* = 0.102
Resting motor threshold (% stimulator output)	43.0 ± 8.6	44.14 ± 11.1	*T* = 0.882, df = 247, *p* = 0.379
Stimulation intensity (% stimulator output)	45.2 ± 8.0	45.1 ± 9.0	*T* = 0.068, df = 247, *p* = 0.946
Number of pulses per session	1989.8 ± 227.6	2002.7 ± 143.3	*T* = 0.451, df = 247, *p* = 0.652
Number of sessions per patient/treatment	17.0 ± 6.3	17.8 ± 6.3	*T* = 0.849, df = 247, *p* = 0.397
HDRS-17 pre treatment	19.4 ± 5.4	19.7 ± 5.8	*T* = 0.518, df = 247, *p* = 0.605
HDRS-21 pre treatment	22.7 ± 6.4	23.3 ± 6.7	*T* = 0.661, df = 247, *p* = 0.509
ICD-10 type of depression (F31/F32/F33)	12/56/106	8/15/50	*χ*^2^ = 3.937, df = 2, *p* = 0.140
ICD-10 severity of depression (mild + moderate/severe/ psychotic)	35/106/6	9/57/6	χ^2^ = 4.995, df = 2, *p* = 0.082
HDRS-17 absolute change	− 7.1 ± 6.3	− 4.5 ± 7.6	*T* = 2.728, df = 247, *p* = 0.007, *d* = 0.372
HDRS-21 absolute change	− 8.1 ± 7.5	− 5.4 ± 8.7	*T* = 2.476, df = 247, *p* = 0.014, *d* = 0.332
HDRS-17 relative change (%)	− 35.7 ± 30.7	− 18.9 ± 42.1	*T* = 3.511, df = 247, *p* < 0.001, 0.456
HDRS-21 relative change (%)	− 34.2 ± 31.2	− 18.8 ± 41.0	*T* = 3.214, df = 247,*p* = 0.001, *d* = 0.423
HDRS-17 relative change > 50% (responder/non-responder)	66/110	13/60	*χ*^2^ = 9.237, df = 1, *p* = 0.002
HDRS item 10 for psychic anxiety	2.1 ± 1.2	2.3 ± 1.1	*T* = 1.313, df = 246, *p* = 0.190
HDRS item 11 for somatic anxiety	1.6 ± 1.0	1.4 ± 1.1	*T* = 1.268, df = 247, *p* = 0.206

*HDRS-17/21* Hamilton depression rating scale 17/21 items; negative values indicate amelioration in HDRS from pre- to post-treatment

## Discussion

In our study, we were able to show that concomitant use of lorazepam significantly reduces the effectiveness of and response rate to rTMS in the treatment of clinical depression with small effect sizes.

This is consistent with theoretical considerations [32] on the interaction between BZD and rTMS, based on the increasing knowledge of the biological basis of MDD [38–40] and the role of rTMS in its treatment [17, 40]. In MDD, clinically depressed individuals show complex dysfunction in gamma-aminobutyric acid (GABA) regulation, including lower GABA levels in cerebrospinal fluid [41] and different parts of the brain [43–47], and normalizing after treatment [48–51]. The therapeutic effect of rTMS is presumably based, among other factors, on the normalization of dysfunctional neural networks [38, 39]. More specifically, the rTMS effect seems not to be based primarily on the intra-network modulation of the target structure itself, i.e., the left DLPFC [17] in MDD, but on an inter-network propagation of the effect to connected neurocircuitries [52, 53], which subsequently influences downstream systems, i.e., neuroendocrine systems such as the hypothalamic–pituitary–adrenal axis [54]. GABA type A receptors (GABA_A_R) seem to be significantly involved in the mediation of this large-scale communication [55]. BZD such as lorazepam are positive allosteric modulators (PAMs) at GABA_A_R [56].

In rodents, down-regulatory effects on GABA signaling, caused by concurrent, chronic use of BZD, have well been studied [57–62] and could tend to mitigate the probable increases in cortical GABA signaling that appear with clinical effective rTMS [50], as it was suggested by Hunter and Minzenberg [29].

In motor cortex studies in healthy individuals, it is well established that this modified behavior of BZD-modulated networks has a significant influence on the effect of rTMS. In experimental models simulating central nervous adaption to peripheral lesions [63] and practice-dependent plasticity [64], single dose of lorazepam reduced cortical plasticity, suggesting that an increase in GABA-related inhibition seems to impede plasticity in the human motor cortex.

In combined TMS-EEG studies, application of the BZD midazolam in anesthetic doses prevented GABA_A_R controlled propagation of TMS-evoked potentials (TEPs) from the stimulation site at premotor cortex to a series of distant cortical areas, as it is normally seen [65]. Similar results were found for TMS stimulation at premotor and parietal cortex after application of anesthetic doses of GABA_A_R PAM propofol [66]. Premoli found that GABA_A_R PAMs alprazolam and diazepam decrease TEP component N100 in the non-stimulated hemisphere [67], which is likely representing GABA_A_ controlled, long-range interhemispheric neurotransmission [55, 65]. These findings suggest that PAMs at GABA_A_R impair the propagation of rTMS effects from the stimulation site to distant brain areas, which may be important for antidepressive efficacy [32, 55].

A clinical rTMS-MRS study showed a trend towards lower increase in medial prefrontal cortex GABA concentration in depressed individuals with chronic lorazepam use [50]. In pooled data with 185 depressed individuals receiving 4 weeks of TMS using different treatment protocols, Fitzgerald et al. found no relationship between benzodiazepine use and clinical response in 64 BZD users compared to 121 patients not taking BZD [68]. Our results are consistent with recent findings of two other large exploratory studies: Kaster et al. [31] found depressed BZD users treated with rTMS to be with reduced odds of membership in a “rapid response” trajectory and with increased odds of membership in a “nonresponse” trajectory and Hunter, Minzenberg et al. [29] found a lower response rate to HF-rTMS applied to the left DLPFC in BZD users. As discussed earlier [32, 69], these findings suggest that BZD may have a negative impact on the antidepressant mechanisms of rTMS, consistent with the results of our and other recently published [29, 31] clinical studies. With three large exploratory studies with 388 [31], 181 [29], and 249 participants in our study, which all arrive at comparable results, there is growing evidence that BZD use is associated with a poorer outcome of rTMS treatment.

There were several limitations to this study. First, it is a retrospective study in a naturalistic setting. Inpatients and outpatients were included. Many of our patients are chronically ill patients, often with multiple psychiatric comorbidities, psychiatric multi-medication and a broad medication history. Even if the two groups with and without BZD did not differ in demographic and clinical characteristics, we cannot exclude that there were differences between the two groups, as there might have been clinical reasons why some of the patients received BZD and others not. However, these reasons seem not to be reflected in the clinical data that are usually recorded even in randomized-controlled trials.

A further important limitation may be that medication, given regularly and on-demand, was recorded exclusively during the study period. This comes with two major limitations: First, the latency of many psychotropic drugs, especially antidepressants, could not be assessed. Second, it was also not possible to determine how long certain drugs, especially BZD were taken and in what doses before the treatment began. This may be especially important, as long-term BZD users tend to shift their use of medication from an as-prescribed to an as-needed pattern [26]. Assuming that several complex control loops with a certain inertia and capacity for self-regulation are involved in the development and chronification as well as in the recovery from depression, the duration of influencing factors is also of decisive importance. Furthermore, medication was not kept stable during the study period and was being changed by the treating physicians in most patients, often to a considerable extent. The intake was not monitored as standard. Serum-level controls were not carried out as standard. Particularly in outpatients, there is still a lack of information regarding the demand-oriented use of (self-)medication or other substance intake, e.g., OTC drugs and/or alcohol. However, patients with indications of clinically relevant substance use disorders were excluded from the study. Multiple psychiatric medication was the rule rather than the exception, especially for patients receiving lorazepam. Further pharmacodynamic and pharmacokinetic interactions must, therefore, be expected. The evaluation of the patient files was carried out with the greatest care by clinical specialists. Nevertheless, there is naturally a certain diagnostic fuzziness in the differential diagnosis of affective disorders, especially depression, compared to other mental disorders, e.g., personality disorders [70] or psychoses [7]. Further limitations are due to high heterogeneity in our naturalistic sample (different types of depression and different treatment protocols).

In our naturalistic study, we found no correlation between lorazepam dose and efficacy of rTMS treatment, most likely due to limitations in study design. Whether such a correlation exists should be further investigated.

It was suggested that the negative influence of BZD on outcome of rTMS is most severe in the first weeks of rTMS treatment [69]. Analyzing improvement of symptom severity in weeks 2, 4, and 6, Hunter, Minzenberg et al. found the largest deficit in BZD users in week 2 [29], while Kaster et al. reported that BZD users were underrepresented in rapid response group during a 4 week course of rTMS treatment [31]. Due to study design, we cannot make any conclusions about trajectories.

## Conclusion

Our results show a reduced effectiveness of rTMS treatment of depression when lorazepam is administered concomitantly. The impact of lorazepam and other BZD on rTMS should receive more attention and should be further investigated in prospective, hypothesis-based treatment studies to determine causal relationships between medication treatments and outcome. Given our results, one should investigate whether patients treated with rTMS for depression may benefit from discontinuation of BZD in controlled trials. Whether the results for lorazepam are also valid for other BZD and the influence of lorazepam dose on efficacy of rTMS should be further investigated. A better understanding of the interactions between rTMS and drugs is important for efforts in increasing the effectiveness of rTMS.

## Data Availability

Not possible due to restrictions of the ethics committee.
